# Common mental disorders and associated factors among adult chronic kidney disease patients attending referral hospitals in Amhara Regional State

**DOI:** 10.1038/s41598-024-57512-1

**Published:** 2024-03-21

**Authors:** Yibeltal Yismaw Gela, Winta Tesfaye, Mihret Melese, Mihret Getnet, Adugnaw Ambelu, Habitu Birhan Eshetu, Desalegn Anmut Bitew, Mengistie Diress

**Affiliations:** 1 Department of Physiology, College of Medicine and Health Sciences, University of Gondar, Gondar, Ethiopia; 2 Department of Health Education and Behavioral Sciences, University of Gondar, Gondar, Ethiopia; 3 Department of Reproductive Health, College of Medicine and Health Sciences, University of Gondar, Gondar, Ethiopia

**Keywords:** Common mental disorders, Chronic kidney disease, SRQ-F, Neuroscience, Physiology, Psychology, Medical research

## Abstract

Common mental disorders (CMDs) are a wide term that includes disorders like depression, anxiety, and somatic manifestations. Chronic kidney disease (CKD) patients are at high risk of developing a common mental disorders, which leads to a lower survival rate, poor clinical outcome, longer hospitalization, increased health-care utilization, difficulty adhering to medications, an increased risk of initiation of dialysis, poor quality of life, and an increased risk of mortality. However, there is limited study done related to common mental disorders and associated factors among chronic kidney disease patients in Ethiopia. This study aimed to assess the prevalence of common mental disorders and associated factors among chronic kidney disease patients attending referral hospitals in Amhara Regional State. An institution-based cross-sectional study design was conducted at the University of Gondar Comprehensive Specialized and Felege Hiwot Referral Hospitals from January to April 2020. The study participants were selected using systematic random sampling techniques. Common mental disorders were assessed using the Self-Reporting Questionnaire-Falk Institute (SRQ-F) tool. Data were entered into Epi Data Version 3.0 then exported into STATA 14 for analysis. Both bivariable and multi-variable binary logistic regressions were done to identify factors associated with common mental disorders. In multivariable logistic regression analysis, variables with a *p*-value of ≤ 0.05 were declared as a statistically associated with common mental disorders. In this study, 424 CKD patients were included, with a response rate of 100%. Among screened CKD patients, 40.8% was positive for common mental disorders, with a 95% CI (36–45%). Independent predictors of common mental disorders among CKD patients were poor social support [(AOR 3.1, 95% CI (1.67–5.77)], family history of mental disorders, [AOR 3.6, 95% CI (1.12–11.8)], comorbidity [AOR 1.7, 95% CI (1.03–2.78)], being female [AOR 2.69, 95% CI (1.72–4.20)], and duration of CKD (AOR 3.5; 95% CI (2.28–5.54). Two out of five CKD patients screened for CMDs were found to be positive. Common mental disorders were more common among CKD patients with poor social support, a family history of mental disorders, comorbidity, being female, and the duration of CKD. Therefore, screening CKD patients for common mental disorders is recommended.

## Introduction

Common mental disorders (CMDs) encompass a broad range of physical, mental, and social disturbances, including conditions such as cognitive impairment, depression, anxiety, and somatic manifestations^1^. Fatigue, insomnia, forgetfulness, irritability, difficulty concentrating, and somatic complaints are among the symptoms experienced by individuals affected by CMDs, leading to long-term effects on human life^2^. Globally, mental disorders account for 14.3% (8 million each year) of all deaths^3^.

According to 2019 estimates, mental diseases account for 16% of disability-adjusted life years lost worldwide^4^. In low- and middle-income nations, mental diseases account for 25.3% and 33.5% of total disability years, respectively^5^.

Depression, psychological distress, cognitive impairment, and anxiety are common mental disorders that frequently affect CKD patients^6,7^. The prevalence of depression in CKD patients is three to four times higher compared to the general population and two to three times higher compared to other chronic diseases^8^.

The magnitude of mental disorders among chronic renal disease patients was 26.6% in the United States^9^.The prevalence of depression was (29.4%), (35.4%), and (23.7%) in Ethiopia^10^, Ireland^11^, and Nigeria^12^, respectively, among chronic kidney disease patients.

Chronic kidney disease patients with CMDs have a higher risk of hospitalizations^13^, poor quality of life^7^, difficulty adhering to medications^14^, rapid progression to end-stage renal disease (ESRD)^9^, worse quality of emotional well-being, and lastly, mortality^8,15^. The risk of death and hospitalization among CKD patients with mental disorders is 11% to 66% and up to 90%, respectively, relative to CKD patients without common mental disorders^9^.

Currently, among the 500 million individuals experiencing mental disorders, only a small percentage are accessing treatment^1^. The gap between the need for treatment for mental disorders and its provision is wide all over the world. In low- and middle-income countries, 76–85% of individuals with severe mental disorders do not receive any treatment for their mental health issues^5^.

In Ethiopia, despite the limited research on the prevalence of depression and anxiety among chronic kidney disease (CKD) patients, this study is the first to provide insight on the overall prevalence of common mental disorders and associated factors among CKD patients.

## Methods and materials

### Study setting and period

An institution-based cross-sectional study was conducted from February to April 2020 among CKD patients attending the referral hospitals in Amhara Regional State.

### Study population

All adult CKD patients encountered at the University of Gondar Comprehensive Specialized and Felege Hiwot Referral Hospitals during the data collection period were included in the study. All CKD patients undergoing dialysis treatment were excluded from this study.

### Sample size calculation and sampling procedure

The sample size was calculated using the following single proportion formula, N = $\left[ {\frac{{{ }\left( {{\text{Z}}_{{{\upalpha }/2}} } \right)^{2} {\text{Xp}}\left( {1 - {\text{p}}} \right){ }}}{{{\text{d}}^{2} }}} \right]$ = $\left[ {\frac{{\left( {1.96} \right)^{2} {\text{X}}0.5\left( {1 - 0.5} \right){ }}}{{\left( {0.05} \right)^{2} }}} \right]$ = 385, where N: sample size, *p*: estimated prevalence value (50%), d: margin of sampling error tolerated (5%), Z_α/2_ (1.96): critical value at 95% confidence interval of certainty.

After adding 10% of the non-response rate, a total of 424 chronic kidney disease patients were selected.

During the time of data collection, Amhara Regional State had five referral hospitals (University of Gondar Comprehensive Specialized Hospital, Felege Hiwot Hospital, Debre Berhan Comprehensive Specialized Hospital, Dessie Comprehensive Specialized Hospital, and Debre Markos Comprehensive Specialized Hospital). Among these, Felege Hiwot Referral Hospital and the University of Gondar Comprehensive Specialized Hospital were selected using the lottery method.

**Figure 1:**
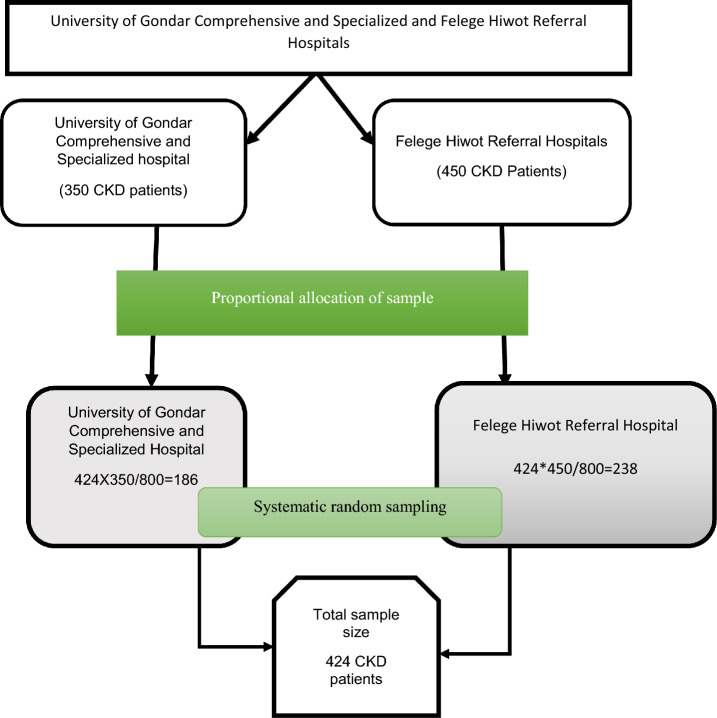
Sampling procedure of chronic kidney disease patients at the University of Gondar Comprehensive Specialized and Felege Hiwot Referral Hospitals, 2020.

Three hundred sixty and four hundred fifty patients were encountered at the follow-up clinics of the University of Gondar Comprehensive Specialized and Felege Hiwot Referral Hospitals, respectively, during the data collection period. Using proportional allocation of sample techniques, 186 and 238 CKD patients were selected using systematic random sampling techniques from the University of Gondar Comprehensive Specialized and Felege Hiwot Referral Hospitals, respectively. A total of 424 CKD patients were recruited using systematic random sampling techniques with a K value of 2. The first participant was the first one selected using the lottery method, and then every second patient was interviewed. To undertake this study, ethical approval was obtained from the Institutional Review Board at the University of Gondar. Additionally, a permission letter was acquired from the University of Gondar Comprehensive Specialized and Felege Hiwot Referral Hospitals. Prior to commencing data collection, written informed consent was obtained from all study participants, ensuring the proper safeguarding of privacy and confidentiality. The research adhered to the principles outlined in the Declaration of Helsinki (Fig. 1).

### Operational definitions

Chronic kidney disease is abnormalities of kidney structure or function, present for > 3 months^16^. The estimated glomerular filtration rate (eGFR) was calculated using epidemiology of collaboration (EPI) Equations^17^.

Common mental disorders (CMDs) were screened using the SRQ-F (Self-Reporting Questionnaire-Falk Institute), which contains 29 items. Those patients who scored ≥ 8 out of 29 scores in the last month were screened as positive for CMD^1,18^.

Social support is assistance provided during times of financial, social, and psychological challenges. The Oslo Social Support Scale tool was utilized to assess social support status, comprising a total of 3 questions scored out of 14. It is categorized as poor support (3–8), moderate support (9–11), and strong support (12–14) based on the scores^19^.

Anemia is defined as a hemoglobin concentration below 13 g/dl in men and below 12 g/dl in women^20^. A current substance user is defined as someone using at least one specific substance within the last 3 months (alcohol, khat, and cigarettes)^21^.

Comorbidity refers to the existence of one or more of the following conditions: HIV/AIDS, hypertension, cardiovascular diseases, and diabetes mellitus.

Individuals with a body mass index (BMI) of (≤ 18.5 kg/m^2^), (18.5–24.9 kg/m^2^), and (25–29.9 kg/m^2^) were categorized as underweight, normal, and overweight, respectively. A BMI exceeding 30 kg/m^2^ was classified as obesity^22,23^.

### Data collection procedure and tools

Data were collected through a face-to-face administration of a structured questionnaire, which consisted of sociodemographic characteristics, substance use history, medical record review, physical measurements (weight and height), the Self-Reporting Questionnaire-Falk Institute (SRQ-F), and the Oslo Social Support Scale.

The Self-Reporting Questionnaire-Falk Institute (SRQ-F) is an instrument comprising 29 questions designed to screen for common mental disorders within the context of Ethiopian culture. Adapted from the SRQ tool, originally developed by the World Health Organization (WHO), this tool aims to assess various aspects, including general health, psychological symptoms, somatic symptoms, anxiety, nervousness, cognitive disturbances, negative self-evaluation, loss of interest in daily activities, and suicidal thoughts. Each of the 29 yes-or-no question items is assigned a score of either 0 or 1, indicating the absence (0) or presence (1) of the symptom over the past 4 weeks. Individuals scoring 8 and above are considered to have screened positively for common mental disorders^2^. The SRQ-F tool is a validated tool in Ethiopia that demonstrates a specificity and sensitivity of 86% and 84%, respectively, within the Ethiopian cultural context, with a Cronbach's alpha coefficient of 0.92^18^.

To compute the body mass index, participants' height and weight were measured with a height measurement stand and weighed using a weighing machine. The estimated glomerular filtration rate (eGFR) was calculated using the 2021 CKD-EPI creatinine equation, the recommended method for predicting eGFR in adults aged 18 years and above^17^. The latest recorded creatinine and hemoglobin values of the study participants were extracted from their medical records.

The Oslo Social Support Scale (OSSS-3) is a self-report questionnaire measuring perceived social support, focusing on the availability of help from friends or neighbors and the ease of obtaining practical or emotional support^19^.

### Data analysis procedure

The data were cleaned, coded, and entered into Epi-Data 3, then exported to STATA 14 for analysis. Continuous variables were presented using the mean and standard deviation, while categorical variables were presented using frequency and pie charts.

Both bi-variable and multi-variable logistic regression analyses were done. The bi-variable logistic regression variables (marital status, income level, social support, sex, eGFR, duration of CKD, family history, and comorbidity) associated with common mental disorders at a *p*-value of ≤ 0.25 were included in the multi-variable regression model. In the multi-variable logistic regression, variables having a *p*-value of ≤ 0.05 with a 95% confidence interval were declared as significantly associated with common mental disorders. Finally, model fitness was checked by the Hosmer and Lemshow test, and it was 0.12 greater than 0.05, the model was fitted. Cronbach’s alpha test for the self-reporting questionnaire-Falk Institute was done with a reliability coefficient value of 0.86, indicating good reliability of the tool.

### Data quality management

The questionnaire was translated to Amharic by a language expert and retranslated back to English by another expert for its consistency. A training regard to the SRQ-F tool, the Oslo Social Support Scale, medical record review, and height and weight measuring procedures was given by the principal investigator for the data collectors. One week before data collection, the questionnaire was pretested at the Tibebe Ghion Specialized Hospital. Based on the pretest findings, the questionnaire was modified.

## Results

### Sociodemographic characteristic of study participants

This research included 424 participants diagnosed with chronic kidney disease, all of whom provided responses, resulting in a 100% response rate. The average age of the study participants was 53.8 years, with a standard deviation of 16.8. The predominant demographic characteristics among the CKD patients were as follows: 60.4% were male, 41.6% attended primary school, 52.5% were employed, 75.7% identified as orthodox Christianity followers, 78.5% were married, and 4.7% reported current substance use (Table 1).

**Table 1:** Socio-demographic characteristics of chronic kidney disease patients at the University of Gondar Comprehensive Specialized and Felege Hiwot Referral Hospitals, 2020.

Variables	Categories	Number (%)
Age (year)	≤ 50	152 (35.9)
	≥ 50	272 (64.1)
Sex	Male	256 (60.4)
	Female	168 (39.6)
Religion	Orthodox	321 (75.7)
	Muslim	72 (17)
	Protestant	31 (7.3)
Occupation	Employed	222(52.5)
	Merchant	71 (16.7)
	Farmer	71 (17.5)
	Housewife	57 (13.2)
Educational level	Unable to read and write	88 (20.7)
	≤ 8 grade	177 (41.6)
	Grade 9–12	72 (17)
	College and above	87 (20.5)
Marital status	Single	54 (12.7)
	Married	333 (78.5)
	Divorced	18 (4.3)
	Widowed	19 (4.5)
Income (ETB)	≤ 1500	130 (30.7)
	1501–3500	107 (25.2)
	≥ 3501	187 (44.1)
Residence	Urban	335 (79.0)
	Rural	89 (21)
BMI (Kg/m^2^)	Normal	345 (81.4)
	Underweight	42 (9.9)
	Overweight	37 (8.7)

*BMI* Body mass index, *ETB* Ethiopia birr.

### Clinical and psychosocial related factors of CKD patients

The mean creatinine level among CKD patients was 1.8 mg/dl (SD ± 1.4). Two hundred thirty-four (55%) patients had eGFR ≥ 90 ml/min/m^2^, and 111 (26.2%) of them were anemic. At the time of data collection, 56% of CKD patients’ duration since diagnosis was 5 years or less. Two hundred ninety-one (68.6%) CKD patients had comorbidities (Table 2).

**Table 2:** Clinical characteristics of chronic kidney disease patients at the University of Gondar Comprehensive Specialized and Felege Hiwot Referral Hospitals, 2020.

Variables	Categories	Number (%)
Creatinine(mg/dl)(Mean ± SD)	1.8	1.8 ± 1.4
eGFR (ml/min/m^2^)	≥ 90	234 (55.2)
	60–89.9	92 (21.7)
	30–59.9	87 (20.5)
	≤ 30	11 (2.6)
Anemia	Yes	111 (26.2)
	No	313 (73.8)
Social support	Poor social support	189 (44.6)
	Moderate support	144 (34)
	Strong support	91 (21.5)
Living status	With family	360 (84.9)
	Alone	64 (15.1)
Duration of CKD (year)	≤ 5 years	229 (54)
	> 5 years	195 (46)
Comorbidity	No	133 (31.4)
	Yes	291 (68.6)

*eGFR* estimated glomerular filtration rate, *CKD* chronic kidney disease.

### Prevalence of common mental disorders among CKD patients

**Figure 2:**
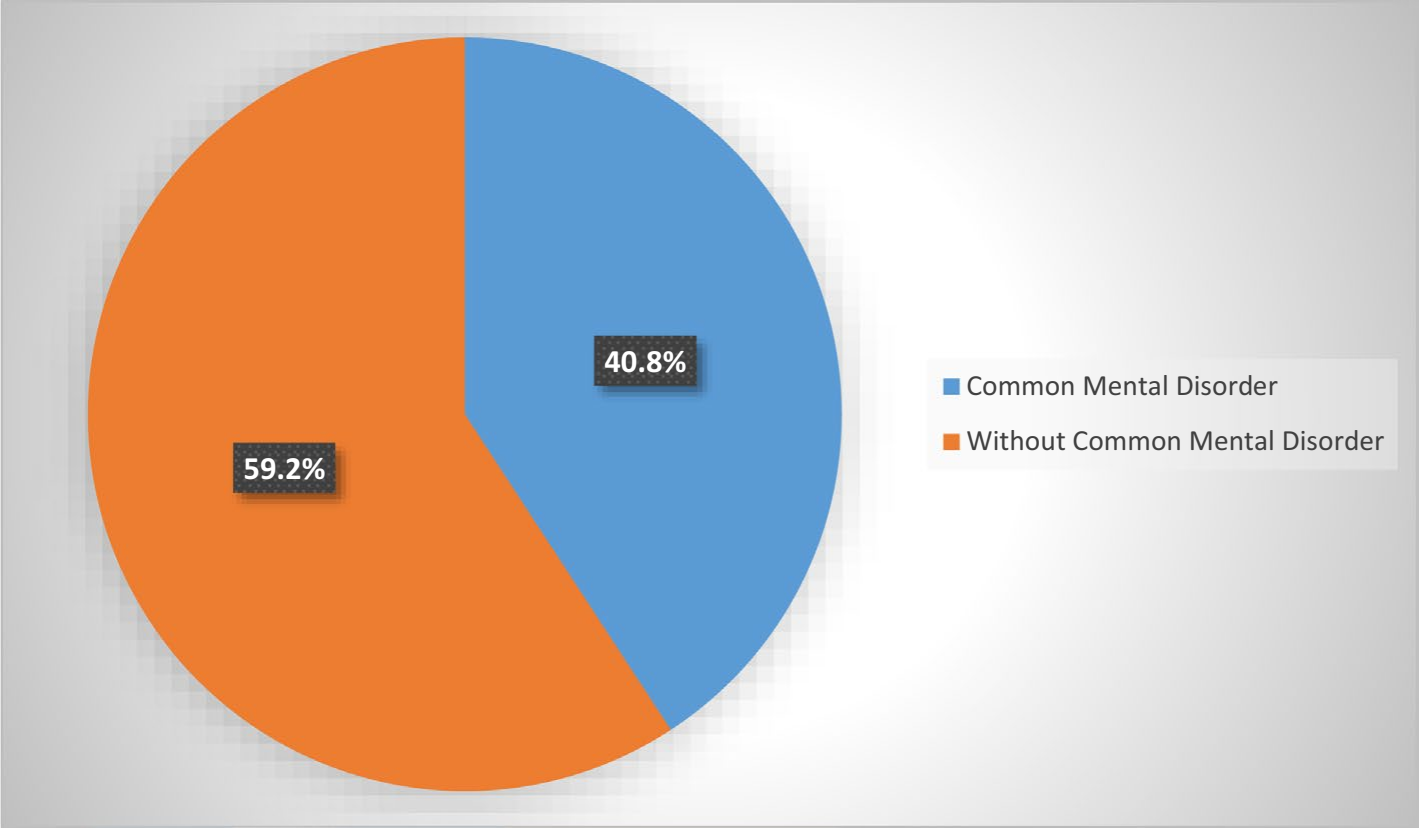
Prevalence of common mental disorders among chronic kidney disease patients at the University of Gondar Comprehensive Specialized and Felege Hiwot Referral Hospitals in 2020.

Among the CKD patients screened for common mental disorders using the SRQ-F tool, 173 (40.8%) tested positive, with a 95% CI (36–45% (Fig. 2).

### Predictors of common mental disorders among CKD patients

Among variables entered into bivariable logistic regression, marital status, income level, social support, sex, eGFR, duration of CKD, family history of mental illness, and comorbidity were associated with common mental disorders at a *p*-value of ≤ 0.25. However, in multi-variable logistic regression analysis, social support, sex, duration of CKD, family history of mental illness, and comorbidity were variables significantly associated with common mental disorders at a *p*-value of ≤ 0.05.

Chronic kidney disease patients with poor social support were 3.1 times [AOR 3.1, 95% CI (1.67–5.77)] more likely to develop common mental disorders than those CKD patients with strong social support. Chronic kidney patients with a known family history of mental disorders were 3.64 times [AOR 3.64, 95% CI (1.12–11.8)] more likely to develop common mental disorders than their counterparts. Chronic kidney disease patients with comorbidities were 1.7 times [AOR 1.7, 95% CI (1.03–2.78)] more likely to develop common mental disorders relative to CKD patients without comorbidities. Those female CKD patients were 2.7 times [AOR 2.69, 95% CI (1.72–4.20)] more likely to develop common mental disorders than those male CKD patients. CKD patients with durations above 5 years were 3.5 times more likely to develop CMDs as compared with those with durations ≤ 5 years (AOR 3.5, 95% CI (2.28–5.54) (Table 3).

**Table 3:** Factors associated with common mental disorders among CKD patients at the University of Gondar Comprehensive Specialized and Felege Hiwot Referral Hospitals, 2020.

Variables	Categories	Common mental disorders			OR (95% CI)	
		Total	Yes	No	COR	AOR
		(N) %	(N) %	(N) %		
Known family history of mental disorders	No	409 (96.5)	164 (38.7)	245 (57.8)	1	1
	Yes	15 (3.5)	9 (2.1)	6 (1.4)	2.24 (0.78–6.4)	3.64 (1.12–11.8)*
Comorbidity	No	133 (31.4)	44 (10.4)	89 (21)	1	1
	Yes	291 (68.6)	129 (30.4)	162 (38.2)	1.6 (1.05–2.47)	1.7 (1.03–2.78)*
eGFR (ml/min/m^2^)	> = 90	234 (55.2)	103 (24.3)	131 (31)	1	1
	60–89.9	92 (21.7)	37 (8.7)	55 (13)	0.85 (0.52–1.39)	0.81 (0.46–1.44)
	30–59.9	88 (20.5)	28 (6.6)	59 (13.9)	0.60 (0.36–1.01)	0.75 (0.41–1.40)
	≤ 30	11 (2.6)	5 (1.2)	6 (1.4)	1.06 (0.31–3.57)	1.67 (0.45–6.23)
Duration of CKD (Year)	≤ 5	229 (54)	62 (14.6)	167 (39.4)	1	1
	> 5	195 (46)	111 (26.2)	84 (19.8)	3.56 (2.37–5.34)	3.5 (2.28–5.54)*
Social support	Poor	189 (44.6)	96 (22.6)	93 (21.93)	0.87 (0.37–2.04)	3.1 (1.67–5.77)*
	Moderate	144 (34)	53 (12.5)	91 (21.5)	1.14 (0.57–2.29)	0.76 (0.84–2.97)*
	Strong	91 (21.4)	24 (5.6)	67 (15.8)	1	1
Sex	Male	256 (60.4)	78 (18.4)	178 (42)	1	1
	Female	168 (39.6)	95 (22.4)	73 (17.2)	2.96 (1.98–4.45)	2.69 (1.72–4.20)*
Marital status	Single	54 (11.7)	19 (10.1)	35 (1.6)	0.82 (0.45–1.48)	1.15 (0.56–2.36)
	Married	333 (78.5)	133 (31.4)	200 (47.1)	1	1
	Divorced	18 (4.7)	9 (2.1)	5 (2.6)	2.36 (0.89–6.25)	1.97 (0.65–5.95)
	Widowed	19 (3.3)	13 (3.1)	7 (0.2)	1.67 (0.66–4.22)	0.94 (0.33–2.73)
Income level (ETB)	> 3500	187 (44.1)	70 (16.5)	117 (27.6)	1	1
	≤ 1500	120 (28.3)	48 (11.3)	72 (17)	1.11 (0.69–1.78)	0.94 (0.55–1.61)
	1501–3500	117 (27.6)	55 (13)	62 (14.6)	1.48 (0.92–2.36)	1.29 (0.74–2.23)

*eGFR* estimated glomerular filtration rate, *CKD* chronic kidney disease, *CI* confidence interval, *COR* crude odds ratio, *eGFR* estimated glomerular filtration rate, *OR* odds ratio, *N* number, *p value ≤ 0.05.

## Discussion

In this study, the prevalence of common mental disorders was 40.8% with a 95% CI (36–45%). It was higher than the study conducted in Korea (28.3%)^24^, Australia (24%)^25^, the United State (26.6%)^9^, and Nigeria (31%)^26^. This might be due to study design, the tool used, and sociodemographic differences. In Korea, the study design was a cohort study. The Diagnostic and Statistical Manual of Mental Disorders, Fourth Edition (DSM-IV) criteria was used for mental disorder diagnosis in both Nigeria and the United States, whereas the Hospital Anxiety and Depression Scale (HADS) was employed in Australia. This study finding was lower than Sri Lanka (65.5%)^27^, Egypt (75.5%)^6^, Tanzania (73.9)^28^,Turky (49%)^29^, Pakistan (65.9%)^30^, Brazil (46.6%)^31^, and Southeast Nigeria (71.2%) studies. This is due to the fact that most of the study participants in Sri Lanka were stage 4 CKD patients, whereas in Egypt, Turkey, Brazil, and Pakistan, the participants were ESKD patients on dialysis. In southeast Nigeria, the mini international neuropsychiatric inventory tool was used.

Those CKD patients with 5-years duration since diagnosis were 3.5 times more likely to develop CMDs as compared with those patients with duration of ≤ 5 years. There are different reasons that explain the association between CKD and common mental disorders. As the duration of the disease increases, uremic toxins accumulation in the cerebrovascular circulation might disturb the normal function of the neural cells^8^. Again, in CKD patients, there is an increase in the levels of inflammatory molecules, reactive oxygen species, and angiotensin II, which may also affect the brain cells, consequently leading to neuropsychiatric comorbidities. Increased levels of interleukin-6, tumor necrosis factor, and interleukin-1β were associated with oxidative DNA damage in brain cells^8^. Generally, in CKD patients, uremia, anemia, hemodynamic changes, sleep disturbance, and hyperparathyroidism are common problems, which explain the link between CKD and neuropsychiatric disorders^8^.

The presence of a known family history of mental disorders was a significant predictor of common mental disorders among CKD patients, which was consistent with other studies^32–34^. The effect of genetic predisposition and the brain structure abnormality that might be transmitted can predispose family members to CMDs^2^. The other possible reason is the effect of stress on caring for mentally ill family members. During this time, the interaction of the caregiver with other people decreases because of stigma and being busy caring for and supporting the family members, which further increases the risk of having CMDs associated with poor social support^2,35^.

The presence of comorbidities in CKD patients was significantly associated with a common mental disorders, similar to other studies^9,36–38^. The presence of comorbidities like diabetes mellitus, hypertension, and cardiovascular disease causes common mental disorders in CKD patients in different ways. Chronic inflammation, changes in hormonal levels, biochemical changes, and polypharmacy were common factors in chronic disease that increased the risk of mental disorders^39–41^. In diabetic mellitus comorbidity, hyperglycemic, hormonal imbalance, and accumulation of free radicals lead to neuronal toxicity and common mental disorders^41,42^. Cardiovascular disease and hypertension can impact mental health through mechanisms such as reduced cerebral blood flow, microvascular damage, white matter lesions, neurotransmitter level disturbance, and the psychological impact of a life-threatening illness^43,44^.

Another factor associated with CMDs was poor social support, which was supported by studies conducted in Addis Ababa^45^, Singapore^46^, China^35^, and Turkey^47^. Inadequate social support has a variety of effects on CKD patients' mental health. These include a lower quality of life, less adherence to recommended treatments, and restricted access to healthcare. Among CKD patients, feelings of isolation, loneliness, and powerlessness as a result of inadequate social support are risk factors for depression and anxiety. Such noncompliance is associated with a rapid deterioration of renal function, which impacts mental well-being^48,49^. The absence of close friends who share the day-to-day stress among CKD patients might result in the occurrence of CMDs, specifically depression, psychological distress, and anxiety in patients with poor social support^48^. Another justification might also be the effect of changing the physiological homeostasis of the hypothalamic pituitary adrenocortical system, which may decrease genetic and other environmental exposures^2,47^. In addition, poor social support might worsen patients’ negative feelings and further contribute to the development of common mental disorders^10^. Social support also improves an individual’s sense of self-efficacy and leads to more understanding, respect, encouragement, courage, and self-fulfillment, all of which can help an individual maintain relatively stable emotions even under pressure^2,35^.

Being female was significantly associated with common mental disorders, which is supported by studies conducted in the USA and China^35,50^. The increased prevalence of CMDs in females cannot be explained by a single cause and is most likely due to a combination of genetic risk, gene-environment interactions, hormones, physiological stress response, sex hormones, and stress^51,52^. Differences in gender roles, gender-based violence, and poor health-seeking behaviour in females are some of the reasons responsible for the higher prevalence of common mental disorders in females^50,53^.

The results of this study provide valuable insights for healthcare professionals regarding the mental health challenges faced by individuals with chronic kidney disease. Integrating the screening and management of common mental disorders into routine CKD care is essential. This approach aims to enhance the overall quality of life and improve various clinical outcomes for CKD patients.

### Strength and limitation of the study

This study's strength lies in its approach of selecting participants from multiple centers, contributing to a broader and more diverse representation of the population. Additionally, the study employed culturally validated tools for the Ethiopian population to screen for common mental disorders.

The SRQ-F instrument was designed primarily for screening common mental disorders and does not provide a definite diagnosis. This tool is incapable of detecting particular illnesses such as depression, anxiety, and cognitive impairment. It is crucial to highlight that questionnaire replies might be influenced by subjectivity and recall bias, thus the results should be interpreted with caution. The SRQ-F's primary objective is to identify individuals who may require additional examination or intervention for mental health difficulties, rather than to provide definitive diagnostic information.

## Conclusions

Two out of five CKD patients screened for CMDs were found to be positive. Common mental disorders were more common among CKD patients with poor social support, a family history of mental disorders, comorbidity, and being female. Therefore, routine screening of CKD patients for common mental disorders and follow-up is recommended.

## Data Availability

The data will be available from the corresponding author upon request.

## Abbreviations

BMI: Body mass index
CKD: Chronic kidney disease
CMDs: Common mental disorders
CVD: Cardiovascular disease
DM: Diabetes mellitus
eGFR: Estimated glomerular filtration rate
GFR: Glomerular filtration rate
